# Clients’ experiences of the Boston Psychiatric Rehabilitation Approach: A qualitative study

**DOI:** 10.3402/qhw.v9.22916

**Published:** 2014-04-08

**Authors:** Henrika Jormfeldt, Bengt Svensson, Lars Hansson, Petra Svedberg

**Affiliations:** 1 School of Social and Health Sciences, Halmstad University, Halmstad, Sweden; 2 Department of Health Sciences, Lund University, Lund, Sweden

**Keywords:** Boston Psychiatric Rehabilitation Approach, clients’ experiences, person-centeredness, shared decision making, qualitative content analysis

## Abstract

The Boston Psychiatric Rehabilitation Approach (BPR) is person-centered and characterized by being based entirely on the individual's unique needs and preferences in the areas of working, learning, social contacts, and living environment. Nevertheless, the person-centered approach is lacking firm evidence regarding outcomes, and empirical studies regarding clients’ experiences of this particular model are needed. A qualitative content analysis of 10 transcribed semistructured individual interviews was used to describe and explore clients’ experiences of the BPR during an implementation project in Sweden. The findings from the interviews could be summarized in “A sense of being in communion with self and others” theme, consisting of three categories: increased self-understanding, getting new perspectives, and being in a trusting relationship. The results showed that clients do not always recognize nor are able to verbalize their goals before they have been given the possibility to reflect their thoughts in collaboration with a trusted person. The guidelines of the approach are intended to support the clients’ ability to participate in decision making regarding their own care. More research about efficacy of different rehabilitation approaches and exploration of fidelity to guidelines of rehabilitation programs are required.

The traditional mental healthcare system in Sweden, like in many other counties, has a strong normative orientation, with a main focus on the reduction of psychiatric symptoms and the prevention of relapses (Van Wel & Landsheer, 2011). This indicates an adherence to behavioral norms mandated for successfully obtaining and maintaining needed support such as housing or entry into a community reintegration program (Lovell, Richmond, & Shern, 1993; Shern et al., 2000). The experience of being a patient in a psychiatric context has been described by former patients as being constrained within a structure of control by a “common staff approach” characterized by power and authority (Enarsson, Sandman, & Hellzén, 2011). In contrast, the Boston Psychiatric Rehabilitation Approach (sometimes called Choose–Get–Keep Model, CGK) (BPR) is designed to be structurally continuous and idiographic in orientation and the activities of the caregivers are directed by client-defined needs, goals, and choices about engaging in rehabilitation (Anthony, 1992; Rogers, Anthony, & Farkas, 2006).

The BPR approach is based on the principles and practices of psychiatric rehabilitation developed by Anthony, Howell, and Danley (1983) at Boston University. The approach was developed for people with a diagnosis of severe mental illness as well as evident limitations in residential, vocational, social, or educational role functioning. The BPR has been described as being neither a particular technique nor an intervention but a service within the mental health system (Farkas & Anthony, 2010), which aims to promote recovery and the achievement of a meaningful life, rather than simply supporting adaptation or survival in the community. Thus, the BPR is person-centered and characterized by being based entirely on the individual's unique needs and preferences (Rogers et al., 2006) in the areas of working, learning, social contacts, and accommodation (Anthony, 1992). The BPR has been studied in a few empirical studies from the United States (Rogers, Anthony, Lyass, & Penk, 2006; Shern et al., 2000) and in a few studies from European countries (Gigantesco et al., 2006; Swildens et al., 2011; Van Busschbach & Wiersma, 2002) with varying outcomes (Michon & Van Weeghel, 2010; Rogers et al., 2006). However studies exploring clients’ experiences of this approach are rare.

This study was a part of a 2-year follow-up project designed to evaluate the implementation of the BPR in a Swedish county. The implementation project was based on the BPR and the purpose of the intervention was to support and guide the client to verbalize and achieve his or her own goals in important life areas such as work or occupation, housing, education, and leisure time. The goals and the scheduling of the intervention were shaped in the interaction between the client and his or her keyworker. Distinct and concrete goals and schedules were made up to support the client in achieving a satisfying life situation. The intervention comprises three different phases where the professional and the client together work through: (1) a diagnostic phase including a comprehensive assessment of the client's abilities and resources, assessment of resources in the client's environment, readiness for rehabilitation, and an overall person-centered goal for the rehabilitation; (2) a planning phase including planning for interventions to strengthen skills development and resource development; and (3) an intervention phase focusing on learning and development of personal skills as well as a resource coordination and adjustment to support the patient to achieve his or her goals. All staff at the services where the BPR was implemented had completed training in the overall BPR methodology and had also supervised training in providing the different phases of the rehabilitation process.

Increased knowledge about clients’ experience regarding their rehabilitation is essential to improve the care of persons with severe mental illness in a way that is more human and cost saving. To our knowledge, no empirical studies have been made regarding clients’ experiences of BPR in Sweden. The purpose of this study was to describe and explore clients’ experiences of the BPR.

## Methods

### Design

The design of the study was descriptive and explorative and based on qualitative content analysis, which is a method aiming to provide new knowledge and understandings, as well as a concrete guide to actions (Krippendorff, 2004). Initially qualitative content analyses dealt with manifest content, but over time latent content has also been included. It has been described as an appropriate method for identifying variations in terms of similarities and differences in a text (Graneheim & Lundman, 2004). Even though qualitative content analysis is described as lacking a solid theoretical background (Krippendorff, 2004), the method is often used in healthcare research. Qualitative content analysis is considered as an appropriate method for the actual study because experiences of the BPR may differ from client to client but also involve something that is shared and complete.

### Participants and settings

Ten participants were purposefully selected from the group of 49 clients who had completed the 2-year follow-up evaluation project (Svedberg et al.,^2013^) to attain variation in terms of sex, age, and experiences regarding duration of illness and previous contacts with mental health services. The criteria for inclusion in the project were that the clients were approached with BPR, had a severe mental illness, were older than 18 years of age, had history of at least 24 months of continuous care in the current services, and presented a need for change in their living situations in areas, such as housing, education, work, employment, or recreational activities. The persons included in the interviews were four women and six men, aged between 23 and 43 years; half of the participants were 30 years of age or younger, and half of the participants were older. Three of the clients were diagnosed with ADHD/autism/Asperger, three with affective disorders, two with schizophrenia, one with an eating disorder, and one with post-traumatic stress syndrome. Six municipal services for persons with mental illness and one outpatient specialist psychiatric service that operated according to the BPR in a county in Sweden participated in the evaluation. Two of the six municipal services only provided vocational rehabilitation. The sample represents all of the services investigated in the actual county.

### Data collection

Qualitative interviews were conducted with clients participating in the 2-year follow-up between August 2009 and December 2010 to reveal the clients’ perspectives regarding the process of rehabilitation during the period between August 2007 and December 2010. The interviews started with the question “How would you describe the rehabilitation process and the outcome reached?”. Follow-up questions focused the clients’ experiences of the following themes: ability to function in daily life, trust in staff, and competence among staff as well as clients’ self-determination and goal setting. The questions were intended to facilitate an open discussion regarding the clients’ experiences of the BPR to ensure that no important aspects of these experiences were overlooked. The participants were encouraged to describe their experiences in their own words. The interviews were recorded and carried out by two of the authors (H. J. and P. S.), who had no involvement in the clients’ care or rehabilitation.

### Data analysis

In the qualitative content analysis described by Graneheim and Lundman (2004), the transcribed interview texts are read through a number of times and interpreted step by step. The transcribed interviews were first read through several times to become familiar with the content. The analysis began by finding the meaning units, that is, the constellation of words or statements that communicate the same central meaning through their content. Meaning units containing aspects related to the client's experiences of the BPR were identified. These meaning units were condensed, abstracted, and labeled with a code while still preserving the central meaning. The codes constitute the basis of finding categories by comparing them to each other to note similarities and differences related to the content of the text. A category is defined as a line of an underlying meaning in the text through condensed meaning units and codes. Subcategories illuminate nuances of the essential sense of each category. The analysis was carried out by the main author (H. J.) and the analyses were evaluated by means of discussions between all authors during the analysis process. The final step in the analysis was to find the theme, which describes the entire result and connects all of the categories (Graneheim & Lundman, 2004).

### Ethics

The study was performed in accordance with the World Medical Association Declaration of Helsinki. The respondents were informed about the purpose and the structure of the study before giving their written informed consent. Participation was voluntary and the respondents were informed that they could withdraw from the study at any time. The study was approved by the Regional Ethical Review Board, Lund University, Sweden, Dnr 316/2007.

## Results

A central theme of how clients’ experienced the BPR was formulated as “A sense of being in communion with self and others.” The theme embraced three categories with a somewhat interrelated relation: “Increased self-understanding,” “Getting new perspectives,” and “Being in a trusting relationship.” The categories and subcategories forming the theme “A sense of being in communion with self and others” are given in Table I.

**Table I:** Units of meaning, codes, subcategories, categories, and theme regarding clients’ experiences of the BPR.

Meaning unit	Condensed meaning units	Code	Subcategory	Category	Theme
Well, work more together with a psychologist and set targets and when it's become time also to apply for a course. He helped me to formulate my goals more than just having this diffuse lot. (Participant 8)	He helped me to verbalize my goals more specifically than my usual diffuse thoughts	To follow-up the individual goals throughout the rehabilitation process	To get help to verbalize individual goals	Increased self-understanding	A sense of being in communion with self and others
					
It felt as if I … had a duty to fulfil. Not just having something to do, but what I did here did not just achieve my own goals … Even if I had one of those really terrible days and didn't want to get up and thought that life was worthless, then I still made a contribution by doing something for someone else. (Participant 2)	Although I had a real awful day, I contributed by doing something useful for others.	To focus on real activities	To do something useful		
					
Being as one notices that … as I said…that he knows what he is talking about and he refers to many, both books and things I have read. Because he worked out fairly quickly that I am the sort who likes to find out why they say so, where they've got the information from…. (Participant 7)	He knows what he is talking about and he refers to many books I have read. He learned fast that I am the kind who wants to know the origin of the information he gave me	That your keyworker knows what he is talking about and is able to give you references	To know the origin of information		
					
All these papers and all this work with rehabilitation together with my keyworker gave me hope because I was forced to work intensely with myself … You were forced to think the thought, it created hope and motivation. You could feel frustration and other feelings that you didn't know you had in this job. You're forced to see that you could actually do something you believed you weren't able to influence. You challenge your own thoughts. I've really had use for this in everyday life, not just in the rehabilitation. (Participant 10)	You are forced to realize that you are able to do something that you thought you could not manage to do	To be forced to think constructively helps you feel hope and motivation	To challenge established negative thoughts	Getting new perspectives	
					
I think that it's a lot to do with the fact you have influence on how the rehabilitation is to be done. Because it took a few times before we realized that the best way was that he was to be a sort of sounding board, where he tests ideas and helps you to see things in a way that you wouldn't have done otherwise. And thus gets you to start thinking for yourself. (Participant 4)	He mirrors my ideas and helps me see things in a way that I had not thought about otherwise. It helped me to start thinking in a new way	To get help to see things that you had not thought about otherwise and thus get help to start thinking in a new way	To support positive thinking		
					
Through discussions and that it hasn't been that he sits up there while I'm down here, we've been equals in the room. (Participant 6)	No one is sitting on a pedestal and no one is sitting down there but we have been equal in the room	To be equal in the discussions	To share decision making		
					
I don't want to have anyone else. No stand-ins, nothing. I really trust her, but she's had to go through … At the beginning when I meet people that I'm going to meet often then I have some sort of unconscious testing time, where I forget them, don't care about them. She's shown herself worthy of my trust there. (Participant 1)	I don't want anybody else. No substitute, nothing … . Now I have great trust in her, but she had to pass some trials to earn my trust	To feel great confidence in your keyworker after some trials	To build trust in the keyworker	Being in a trusting relationship	
					
If a person sighs and if you get the feeling that you ought to be ashamed then you don't want to be with them. If you've been mobbed as I have, then you assume that others don't want to be with you. She was personal and it's important, that you feel this warmth between the two of you…. (Participant 3)	She was personal and that is important … to feel the closeness between you and the keyworker	She was personal and that is important, to feel friendliness between you and the keyworker	To like and understand each other		

### Increased self-understanding

The category *increased self-understanding* contains the clients’ experiences of learning to verbalize individual goals, engaging in daily tasks, being useful to others, and realizing one's individual ability by being enforced to think constructively. The manual of the BPR was experienced as important in order for supporting the follow-up process and strengthen the relationship between the keyworker and the client. The category contains three subcategories: to get help to verbalize individual goals, to do something useful, and to know the origin of information.

### To get help to verbalize individual goals

Clients’ experiences of getting help in mirroring their thoughts and in supporting the development of new constructive thoughts was illustrated through the subcategory *to get help to verbalize individual goals*. The support from the keyworker was experienced as fostering the ability to recognize and verbalize individual goals. A crucial experience was the process of becoming familiar with one's goals.

Well, the aim at the start was sort of: “Get well!” There it was: What do you do to get well? …. He helped me to formulate my goals more than just having this diffuse lot. (Participant 8)

The experience of a lack of structure when the manual of BPR was not completed by the keyworker and the continuous nonavailability of help to the client to verbalize and follow-up his or her individual goals were also described.

We had a folder with the old aims and … But she didn't think that we needed to write anything more. Because we knew anyway what we ought to work towards. So it felt … It wasn't very good, because I think that … I think it's very important to talk about goals, as though they are something that can change. (Participant 2)

### To do something useful

The subcategory *to do something useful* addresses the importance of focusing on real activities and being able to contribute by doing something useful for others. Experiences of meaningfulness when contributing to daily activities during rehabilitation were expressed.

It felt as if I … had a duty to fulfil … Even if I had one of those really terrible days and didn't want to get up and thought that life was worthless, then I still made a contribution by doing something for someone else. (Participant 7)

### To know the origin of information

Clients expressed the experience that the keyworker knows what he or she is talking about and is able to give you references. The experience of being informed of the origin of information was conveyed.

Because he worked out fairly quickly that I am the sort who likes to find out why they say so, where they've got the information from …. (Participant 4)

### Getting new perspectives

The structure of the BPR approach is described by clients as securing the continuity in participation regarding goal setting and care planning. The category *getting new perspectives* comprises the clients’ experiences of being respected as equal individuals and the importance of getting the necessary resources and insights to be able to make decisions and set goals according to individual preferences. The category includes three subcategories: to challenge established negative thoughts, to support positive thinking, and to share decision-making.

### To challenge established negative thoughts

The subcategory *to challenge established thoughts* involves the significance of being forced to realize that you are able to do more than you expect to be able to manage. Clients experienced that being forced to think constructively helped them to feel hope and motivation by being challenged in their negative thoughts.

You could feel frustration and other feelings that you didn't know you had in this job. You're forced to see that you could actually do something you believed you weren't able to influence. You challenge your own thoughts. I've really had use for this in everyday life, not just in the rehabilitation. (Participant 10)

### To support positive thinking

Clients expressed the experience of getting help to see things that you had not thought about otherwise and thus getting help to start thinking in a new way. Experiences of the importance of getting ideas mirrored to be able to see things in a new way were described.

…he tests ideas and helps you to see things in a way that you wouldn't have done otherwise. And thus gets you to start thinking for yourself. (Participant 4)

### To share decision making

The subcategory *to share decision making* comprises the experience of being met on an adult level, being listened to, and having the opportunity to choose. Being respected as equal in the decision making was experienced.

…it hasn't been that he sits up there while I'm down here, we've been equals in the room. (Participant 6)

The experience of not shared decision making (SDM) leading to lack of equality and a feeling of being reduced and worthless was also described.

You came here and then they told you what to do: ‘we're cleaning today’ or ‘no, you're not going to sew potholders today, you're going to be there instead’. So we didn't meet at an adult level. No, I don't know. It felt …. (Participant 2)

### Being in a trusting relationship

The category *being in a trusting relationship* embraces the clients’ experiences of feeling confidence in their keyworker. A feeling of a mutual liking of each other and a feeling of being understood by the keyworker was expressed. Confidence was often developed after the dependability of the relationship had been tested. The category comprises two subcategories: to build trust in the keyworker, and to like and understand each other.

### To build trust in the keyworker

Clients’ experiences of how trust was built in the relationship with the keyworker in the BPR approach were central. The subcategory includes the insight that it may take a trial period to build great confidence in the keyworker. This experience of building trust was expressed.

…At the beginning when I meet people that I'm going to meet often then I have some sort of unconscious testing time, where I forget them, don't care about them. She's shown herself worthy of my trust there. (Participant 1)

### To like and understand each other

The subcategory *to like and understand each other* involves the awareness of the importance of the client and the keyworker liking and understanding each other. Negative feelings of being ashamed and reduced when a person sighs and complains about you were expressed. The opposite experience of a valuable and supportive friendship also was described.

If you've been mobbed as I have, then you assume that others don't want to be with you. She was personal and it's important, that you feel this warmth between the two of you …. (Participant 3)

## Discussion

### Discussion of results

The purpose of this study was to describe clients’ experiences of the BPR approach. The analysis of the clients’ experiences resulted in three categories: *increased self-understanding*, *getting new perspectives*, and *being in a trusting relationship* which can be seen as important parts of the theme *a sense of being in communion with self and others*. The categories are interwoven and no absolute boundaries can be found between them; however, it may be beneficial to elucidate each dimension of the clients’ experiences of BPR separately.

The results indicate that the guidelines and manuals of the BPR approach are important and support the rehabilitation process as well as the relationship between the keyworker and the client. The results suggest that the BPR approach incorporates a special structure that the clients perceive as securing their participation and safety. The clients in this study expressed that the structure of the BPR approach gave them opportunities to reflect on their thoughts and to challenge and give up negative thoughts. They also expressed that it made it easier to start acting constructively as well as thinking positively about themselves and their possibilities. When the guidelines of the model were not followed by the keyworker, it reinforced the clients’ inability to take their own initiative. Thus, the findings in this small study regarding usefulness of the structure of a continuous and idiographic orientation directed by client-defined choices in the BPR approach as described by Anthony (1992) could be considered as important in further development of psychiatric rehabilitation approaches.

The findings in this study illustrate the importance of participation in terms of being respected as an equal. The structure of the BPR approach is described by the clients as securing the continuity in participation regarding goal setting and care planning. Taking part in decision-making processes have shown a positive impact on the individual's capability to reflect on old habits in more constructive ways as well as to improve psychosocial functioning (McCann & Clark, 2004). Equality is referred to by the clients as being revealed through a dialogue aiming to support the clients’ personal resources and insights, necessary to be able to make own decisions and set goals in accordance with individual preferences. The findings of the actual study thus indicate that the BPR have solid similarities with person-centered care and SDM. These approaches are based on principles of respect and a partnership with people receiving healthcare (Hamann et al., 2006; Law, Baptiste, & Mills, 1995). One problem regarding implementation of the BPR is that traditional mental healthcare systems often have a main focus on the reduction of psychotic symptoms and the prevention of relapses (Van Wel & Landsheer, 2011). A strict provider-centered approach in mental health services mainly focusing on medical treatment may not automatically increase quality of life and the ability to achieve personal goals among persons with severe mental illness (Chee, 2009). However, healthcare organizations can promote empowerment by implementing programs properly, and ensure that staff members have sufficient time to involve clients’ in treatment planning and stimulate them to support clients’ ability to participate (Linhorst, Hamilton, Young, & Eckert, 2002). Sharing medical decisions with inpatients diagnosed with schizophrenia has resulted in a significantly better knowledge about the diagnosis, a higher level of perceived involvement in medical decisions, and an increased uptake of psychoeducation (Hamann et al., 2006). SDM is, however, in the mental health field, a relatively new and somewhat controversial concept (Forrest, 2004). The client is empowered by being an active participant in the decision-making processes regarding their own care (Linhorst et al., 2002) hence knowledge about the perspective of the client and SDM is a fundamental component of evidence-based medicine. It has been claimed that a truly collaborative care to sufficiently support processes of empowerment for people with mental health problems requires a major redefinition of roles and relationships among healthcare professionals and clients (Anderson & Funnell, 2005). Clients in previous research have shown to be empowered not only by the outcomes of the decisions he or she makes but also by being an active participant in the decision-making process (Linhorst et al., 2002).

The category of being in a trusting relationship embraces the clients’ experiences of feeling confidence in the keyworker, a feeling of being understood, and a mutual liking of each other. These positive feelings were developed after the keyworkers’ dependability had been put on trial. The finding is in line with the reasoning of Farkas and Anthony (2010). A commitment to a strong partnership between the provider and the client is considered as the basis of psychiatric rehabilitation. Trust, choice, and empowerment of patients have previously been depicted as important aspects in psychiatric care but one important problem among the patients has often been their inability to take part in their own treatment (Laugharne & Priebe, 2006). Patients in mental health services have expressed appreciation of the expertise of clinicians, but it has been maintained that they particularly appreciate the personal interaction beyond this expertise, such as mutual acts of kindness and everyday conversation (Laugharne, Priebe, McCabe, Garland, & Clifford, 2012). Also the quantity and quality of time has been considered to be of importance for the construction of the working alliance between the professional and the client (Topor & Denhov, 2012). The findings of the present study suggest that a good relationship between the client and the keyworker is of importance for a successful recovery, but it also might be a result of the rehabilitation process. A good relationship needs to focus on the ingredients within the relationship which actively support the client to lead a successful, dignified life (Browne, Cashin, & Graham, 2012). Nevertheless, a relationship without structure and the possibilities to participate in decision making is not sufficient on its own, and it was not experienced as professional and adequate by the clients in the actual study.

### Methodological considerations

When using qualitative content analysis, it is preferable that the sample has a variation regarding sex, age, and experiences of the studied topic to increase the possibility of the research question being answered from different perspectives (Graneheim & Lundman, 2004). In this study, the included clients were purposely selected in connection with the 2-year follow-up of the evaluation project to attain a variation regarding sex, age, and experiences. The fact that interviewees were collected from a 2-year follow-up evaluation could lead to a less heterogeneous sample and the relatively small sample reduces the transferability of the results to the overall population of clients with severe mental illness using BPR. In qualitative research, it is important that the interviewer and interviewed have a mutual understanding about the topic of the interview to secure the dependability of the data collected. Dependability in this study was strengthened by the questions which were intended to facilitate an open discussion regarding the clients’ experiences of the BPR to ensure that no important aspects of these experiences were overlooked. Dependability was further strengthened by the fact that the interviews were recorded and carried out by two of the authors, who had no involvement in the clients’ care or rehabilitation and the interviewers’ also had extensive knowledge and experiences of the topic of the interview and of interviewing. A detailed description of the process of the analysis in Table I illustrates how the original meaning units have been condensed, abstracted, coded, and categorized. The detailed description increases the possibility for estimating the credibility of the results when using qualitative content analysis (Graneheim & Lundman, 2004). Through the detailed description of the analysis and the fact that the categories were exemplified with unfolding quotations, credibility is regarded as satisfactory.

## Conclusion

The purpose of this study was to describe and explore clients’ experiences of the BPR. The analysis of the clients’ experiences resulted in three categories: increased self-understanding, getting new perspectives, and being in a trusting relationship, which can be seen as important parts of a sense of being in communion with self and others. The clients’ expressed experiences of the BPR approach as an opportunity to recognize and verbalize their individual goals. The findings shows that clients often do not recognize or are not able to verbalize their personal goals before having been given the possibility to reflect on their thoughts in collaboration with a trusted person. The manual of the BPR approach is referred to as securing client participation by regularly giving the clients the opportunity to get their thoughts reflected and to be able to participate in decision making regarding their own rehabilitation.

### Implication

Psychiatric rehabilitation as well as education among mental healthcare professionals should involve person-centered approaches and training in SDM strategies. Implementation of BPR may require a paradigm shift in healthcare organizations to develop a destigmatizing paradigm. Additional research is needed to investigate to what extent the BPR approach systematically improves the clients’ life situation in comparison to other rehabilitation models. Additional research is also desirable regarding involvements of relatives in the rehabilitation to target needs of social involvement among persons with severe mental illness.
